# *Xenopus laevis* neural stem progenitor cells exhibit a transient metabolic shift toward glycolysis during spinal cord regeneration

**DOI:** 10.3389/fcell.2025.1529093

**Published:** 2025-01-29

**Authors:** Paula G. Slater, Miguel E. Domínguez-Romero, Guillermo Campos, Vania Aravena, Javier Cavieres-Lepe, Verónica Eisner

**Affiliations:** ^1^ Laboratory of Neuro-Regeneration and Metabolism, Fundación Ciencia & Vida, Huechuraba, Santiago, Chile; ^2^ Departamento de Ciencias Biológicas y Químicas, Facultad de Medicina y Ciencias, Universidad San Sebastián, Santiago, Chile; ^3^ Facultad de Ciencias Biológicas, Pontificia Universidad Católica de Chile, Santiago, Chile

**Keywords:** mitochondria, glycolitic shift, regeneration, neural stem progenitor cells (NSPCs), metabolic regulation, *Xenopus laevis*

## Abstract

Spinal cord injury (SCI) results in severe disruption of communication between the brain and body, causing motor, sensory, and autonomic dysfunctions. While SCI in mammals leads to permanent impairment due to limited regenerative capacity, certain non-mammalian species, such as *Xenopus laevis* larval stages, exhibit remarkable regenerative abilities. During *Xenopus laevis* spinal cord regeneration, neural stem precursor cells (NSPCs) surrounding the central canal rapidly proliferate in response to SCI, compensating for cellular loss, restoring canal continuity, and generating new neurons to reestablish lost connections. It has been described that mitochondria and cellular metabolism play essential roles in stem cell proliferation, self-renewal, and differentiation. However, the mitochondrial and cellular metabolic response during spinal cord regeneration remains unexplored. This study uses electron and confocal microscopy to investigate the NSPCs mitochondrial response in *Xenopus laevis* following SCI. We observed that mitochondria exhibit a rapid and transient response after SCI, characterized by a disruption of the mitochondrial localization, a decrease in mitochondrial number per cell section, and an increase in mitochondrial area and circularity. Furthermore, mitochondria adopted a swollen phenotype, which did not impair mitochondrial function or cellular energy balance. This morphological shift was accompanied by a transient decrease in the mitochondrial membrane potential and a metabolic switch favoring glycolysis. Therefore, these findings demonstrate that a transient metabolic shift toward glycolysis occurs during spinal cord regeneration.

## Introduction

The spinal cord is part of the central nervous system (CNS), comprised of some nerves responsible for receiving sensory information and others controlling the motor responses of the limbs and trunk muscles (Watson et al., 2009). Spinal Cord Injury (SCI) is a severe traumatic event that interrupts the two-way flow of information between the brain and spinal cord, resulting in paralysis below the area where the damage occurs, generating motor, autonomic, and sensory dysfunction (Thuret et al., 2006). It is estimated that there are between 250.000–500.000 new cases of SCI worldwide every year (Organization and Society, 2013), and due to the inefficient regenerative ability of the mammalian CNS and the absence of therapies allowing functional recovery, this affliction is permanent (Thuret et al., 2006; Organization and Society, 2013).

In mammals, the damage produced by SCI comprises two main phases. The primary injury starts with the initial mechanical insult that disrupts the structural integrity of the tissue and generates a hemostatic response, damage of axons, and death of oligodendrocytes, ultimately leading to tissue functional loss. This is followed by a secondary phase marked by the propagation of the biochemical changes, leading to an extension of the damage both rostrally and caudally from the injury site (Grossman et al., 2001; Quadri et al., 2020). This secondary phase consists of three distinct cellular stages: cell death and inflammation, cell proliferation and tissue replacement, and tissue remodeling (Burda and Sofroniew, 2014). SCI induces excitotoxicity (Park et al., 2004) and free radicals (Liu et al., 2000; Cuzzocrea and Genovese, 2008), leading to neuronal, oligodendrocyte, astrocyte, and neural stem progenitor cells (NSPC) death (Slater et al., 2022a). Pro-inflammatory immune cells are attracted to the injury site to remove cellular debris, which can hinder the regeneration of surviving neurons. However, for effective regeneration to occur, there must be a shift toward an anti-inflammatory phenotype (Gensel and Zhang, 2015; Slater et al., 2022a). Later, the proliferation of oligodendrocyte progenitors, astrocytes, and ependymal cells lining the central canal, which have *in vitro* neural stem cell potential, occurs, generating mostly astrocytes (Meletis et al., 2008; Barnabé-Heider et al., 2010). Finally, a glial scar is formed; this structure safeguards the spinal cord from widespread injury area (Karimi-Abdolrezaee and Billakanti, 2012; Gu et al., 2019), but it hinders effective regeneration (Davies et al., 1997).

Some non-mammalian organisms, including teleost fish, urodele amphibians, and the larval stages of anuran amphibians, possess remarkable regenerative capabilities, enabling them to recover from severe spinal cord injuries (Diaz Quiroz and Echeverri, 2013; Lee-Liu et al., 2017). Understanding the cellular and molecular mechanisms that facilitate successful regeneration in these species is crucial for comparing them with non-regenerative organisms. This comparison can help identify what is lacking in mammals and guide the development of new therapeutic targets. Notably, *Xenopus laevis* possesses a unique capacity to regenerate its spinal cord during larval stages (NF stages 48–52), but this ability is lost after metamorphosis (NF stage 66) (Hooker, 1925; Beattie et al., 1990; Muñoz et al., 2015), making it an excellent model system to study regeneration (Slater et al., 2021; Slater et al., 2022b).

*Xenopus laevis* regenerative stages (R-stages) present extensive NSPCs surrounding the spinal cord central canal (Edwards-Faret et al., 2021) and respond to SCI with a quick increase in proliferation (Muñoz et al., 2015; Peñailillo et al., 2021). Cellular proliferation is needed to compensate for the cellular loss induced by SCI, facilitating the generation of new NSPC-like cells to maintain central canal continuity and produce new neurons that restore lost connections and spinal cord functionality (Edwards-Faret et al., 2021). It has been described that mitochondria and cellular metabolism play important roles in stem cell proliferation, self-renewal, and differentiation. Usually, stem cells contain a low number of mitochondria and depend mostly on glycolytic metabolism (Rafalski et al., 2012). Cells with high proliferative rate are characterized by a metabolic switch favoring glycolytic metabolism (DeBerardinis and Chandel, 2020), which is necessary for activating anabolic pathways for the generation of cellular building blocks (Liberti and Locasale, 2016), but also for remodeling cell cycle regulatory proteins (Liu et al., 2023). After stem cell proliferation, mitochondria and cellular metabolism determine whether a cell will remain a stem cell or undergo differentiation. A cell that inherits newly formed mitochondria and maintains a glycolytic metabolism continues as a stem cell (Zhang et al., 2018). In contrast, a cell that inherits older mitochondria and shifts its metabolism toward oxidative phosphorylation is the one that differentiates (Yanes et al., 2010; Zheng et al., 2016). Additionally, high throughput experiments analyzing the *Xenopus laevis* transcriptomic response at different time points after SCI in R-stage animals, showed that the highest number of transcripts differentially regulated was at 1-day post transection (dpt) and more than 50% of transcripts that respond differentially, were of genes involved in metabolic processes (Lee-Liu et al., 2014). Still, no previous studies have examined the mitochondrial adaptive response associated with spinal cord regeneration.

This study employed electron and confocal microscopy to characterize the temporal mitochondrial response in wild-type and transgenic *Xenopus laevis* lines following SCI. Herein, we described that mitochondria present a rapid response after SCI. Mitochondria are expressed mainly in the apical region of the NSPCs surrounding the spinal cord central canal, a disposition that is lost after SCI. Additionally, a decrease in the number of mitochondria per cell section is observed, accompanied by an increase in the mitochondrial area and circularity. This change, it seems, is not highly regulated by adaptations in mitochondrial fusion- and fission-related genes. SCI also induces a phenotypic change in mitochondrial morphology, with a preference for a swollen morphology, which does not compromise mitochondrial function. Furthermore, this morphological change is accompanied by a transient decrease in NSPCs mitochondrial membrane potential and a shift toward glycolytic metabolism, showing that a transient metabolic shift toward glycolysis might be necessary during spinal cord regeneration. Therefore, these results show mitochondrial adaptation and metabolic reprogramming during regenerative processes and provide valuable information to be used as a starting point to define possible new therapeutic targets to promote CNS regeneration.

## Materials and methods

### Animal husbandry

*Xenopus laevis* embryos were obtained by natural mating of wild-type male and female frogs. Animal husbandry was performed as previously described (Edwards-Faret et al., 2017; Slater and Larraín, 2021). Animals were grown up until Nieuwkoop and Faber stages 50–51 (NF 50–51) (R-stages) for experiments (Nieuwkoop, 1994). All animal procedures were approved by the Scientific Ethics Committee for the Care of Animals and Environment of the Pontificia Universidad Católica de Chile and Bioethical and Biosafety Committee of Universidad San Sebastián (Protocol 181017006 and 210504022).

### Spinal cord injury

R-animals were anesthetized with 0.02% of tricaine mesylate (MS222) dissolved in 0,1x Barth solution (8.9 mM NaCl; 102 μM KCl; 238.1 μM NaHCO_3_; 1 mM 4-(2- hydroxyethyl)-1-piperazine ethanesulfonic acid (HEPES); 81.14 μM MgSO_4_; 33.88 μM Ca(NO_3_)_2_; 40.81 μM CaCl_2_, pH 7.6). Spinal cord transection was performed as previously described (Edwards-Faret et al., 2017; Slater and Larraín, 2021). Briefly, the skin and dorsal muscles were opened at the mid-thoracic level, and the spinal cord was fully transected with a clean cut at the thoracic level with micro-spring scissors. After surgery, animals were transferred into their tanks with 0.1 x Barth supplemented with antibiotics (penicillin and streptomycin).

### Transmission electron microscopy

For transmission electron microscopy (TEM) analysis, uninjured and transected animals (6 h post-transecting (hpt), 1dpt, and 2dpt) were fixed with a 2% PFA and 2.5% glutaraldehyde solution at 4 C overnight. Spinal cords were processed by the Advanced Microscopy Facility UMA-UC, Pontificia Universidad Católica de Chile. Images of ultra-thin sections were acquired in a transmission electron microscope Philips Tecnai 12 at 80 kV, at the Advanced Microscopy Facility UMA-UC, Pontificia Universidad Católica de Chile. Three independent experiments were performed.

### Spinal cord cells dissociation

Uninjured animals and animals at 6hpt, 1dpt and 2dpt were anesthetized in 0.02% MS222. The entire caudal segment of the spinal cord, from 10 to 15 animals, was isolated from the injury site with forceps and collected in 1X Marc’s Modified Ringer solution (MMR) (0.1 M NaCl, 2.0 mM KCl, 1.0 mM MgSO4, 2.0 mM CaCl2, 5.0 mM HEPES, pH 7.4). To disaggregate the tissue, the spinal cords were incubated in 100 uL of 1X papain in 1X MMR–Ca^2+^, Mg^2+^ free (1X MMR–CMF) for 10 min, homogenizing with a pipette every 5 min. The papain reaction was stopped with the same volume (100 uL) of Fetal Bovine Serum. Later, the samples were centrifuged at 1,000 g for 5 min, and the cells were resuspended in 100 uL of 1X MMR - CMF. To quantify the cells, a solution with trypan blue was prepared at a 1:10 dilution and the cells were quantified in a Neubauer chamber under a light microscope at 10 X magnification.

### Mitochondrial membrane potential

The spinal cord of EGFP + animals at R-stage from the transgenic line zGFAP::EGFP, expressing EGFP specifically in NSPCs (Edwards-Faret et al., 2021), was transected. The spinal cord cells dissociated at 6 hpt, 1dpt, 2dpt and from uninjured animals (15 animals per condition). Glass coverslips were coated with 100 µg/mL Poly-L-lysine for 1 h at 37°C, and 50 µL of dissociated cells were seeded. After 5 min, the cells were loaded for 10 min at room temperature (RT) with 20 nM Tetramethyl rhodamine ethyl ester (TMRE) probe, and the cell media was changed for a media containing 0.25% bovine serum albumin and 0.2 nM TMRE. Images were obtained at the Nikon Eclipse Ti microscope using the following configuration: first, a single image was obtained using ex. 488 nm—em. 500/550 nm for detecting FGP positive cells, and ex. 540 nm—em. 620/60 nm for detecting TMRE. Later, it was obtained 1 image every 1.5 s for 5 min, using ex. 540 nm—em. 620/60 nm. 10 μM FCCP was used to induce loss of mitochondrial membrane potential (ΔΨ). Resting membrane potential was calculated as ΔF_basal_—F_FCCP_ using absolute fluorescence values.

### Phosphofructokinase activity assay

Phosphofructokinase (PFK) activity was measured with colorimetric kits (MAK093, Sigma; ab155898, Abcam) following the supplier’s protocol. Briefly, spinal cord cells were dissociated at 6 hpt, 1dpt, and 2dpt, and from uninjured animals (15 animals per condition), and between 500,000 and 1,000,000 cells were centrifuged at 1,000 g for 5 min, the supernatant was discarded, and cells resuspended in 100 µL cold assay buffer. After pipette homogenization, samples were incubated on ice for 10 min and centrifuged at 12,000 g for 5 min at 4°C, and the equivalent of 10,000 cells was added to designated wells together with the reaction mix. Readings were taken in a Synergy H1 Hybrid Reader (Biotek) preheated to 37°C, with measurements every 5 min at 450 nm for 20–60 min. Three independent experiments were performed.

### Mesurements of ATP levels

ATP levels were measured with CellTiter-Glo™ 2.0 Assay (Promega) following the supplier’s protocol. Briefly, spinal cord cells were dissociated, and 80,000 cells were added to the designated wells together with the reaction mix. Luminescence readings were obtained in an Infinite M200 pro (Tecan). Four independent experiments were performed. Oligomycin was used as a control.

### Immunofluorescence

Uninjured animals were sacrificed and fixed in 4% paraformaldehyde for 2 h at RT or overnight at 4°C. The samples were dehydrated with a sucrose gradient, cryoprotected in optimal cutting temperature compound, and frozen with liquid nitrogen. Longitudinal and transversal 10 µm sections were obtained using a Leica CM1850 cryostat. Samples were permeabilized in 1X phosphate-buffered saline (PBS) with 0.2% Tritonx-100 (PBST) for 10 min and blocked with 10% goat serum in PBST for 30 min. Later, the samples were incubated with the primary antibodies at 4°C overnight and secondary antibodies for 2 h at RT; after each antibody incubation, samples were washed 3 times for 10 min with PBST, followed by nuclei staining with Hoechst solution (1:10,000) for 5 min and mounted with Vectashield (H-1000-NB). Epifluorescence images were taken in an Olympus microfluo microscope IX71. Rabbit anti-COXIV (1:1000, Abcam, ab16056) was used to detect mitochondria, Mouse anti-SOX2 (1:200, Cell Signaling Techonology, 2748 S) was used to detect NSPCs, and rabbit anti-NeuN (1:1000, SDIX) was used to detect mature neurons. The secondary antibodies donkey anti-mouse AlexaFluor^®^ 488 and donkey anti-rabbit AlexaFluor^®^ 594 (1:500, Jackson Immuno Research), goat anti-rabbit AlexaFluor^®^ 488 and goat ant-mouse AlexaFluor^®^ 594 (1:500, Invitrogen). Nuclei were stained with Hoechst (1:10.000) and mounted with vectashield (Vector Laboratories, H-1000).

### RT-qPCR

Uninjured animals and animals at 2, 6, and 10 hpt, and 1 and 2dpt were anesthetized in 0.02% MS222. The entire caudal segment of the spinal cord, from 10 to 15 animals, was isolated from the injury site with forceps, and total RNA was obtained with the commercial kit (RNeasy Mini Kit) following the supplier’s protocol. RNA concentration was measured using Nanodrop (Thermo Scientific). The cDNA was synthesized using the M-MLV reverse transcriptase (Promega), and qPCR was performed using Power SYBR Green (Applied Biosystems) or Maxima SYBR Green (Thermo Scientific) by performing three technical replicates. The relative expression ratio was then calculated as described (Pfaffl, 2001), using *eef1a1* (GenBank: BC043843) as the reference gene. The primers used were *mfn2, opa1, mtfr, mtfp, hk2, pfkfb1,* and *pklr*. For the sequence of the primer, please see Table 1.

**TABLE 1:** Primers used for RT-qPCR and their corresponding sequences.

Gene	Primers
mnf2	Fw: 5′TTT​CCT​TTG​ACA​TGA​TGC​TG 3′
	Rv: 5′ACT​CAA​TGA​TCT​GAT​TTT​GGA​AGA 3′
opa1	Fw:5′TGCAAACACTCTGAGACAGCA 3′
	Rv 5′TTA​TCA​CCG​TCT​TCG​GCA​AA 3′
mtfr	Fw:5′AGCCAAGCCTGAATATGGAC 3′
	Rv 5′GCT​TCT​GTA​GCA​TTG​GAT​AAT​GA 3′
mtfp	Fw:5′GAGCACTAGTTCCCAAAGCAG 3′
	Rv 5′AGA​ATT​TGC​TGC​TTT​CTT​GC 3′
hk2	Fw:5′TGACCAGAGATCTATTCCCTACC 3′
	Rv 5′GGA​GGC​CGA​GCT​CTG​TTA​G 3′
pfkfb1	Fw:5′TACCACCGCTGCCTATCAAT 3′
	Rv 5′GGA​GAT​ATA​AGT​CTT​CCC​TCT​TGC 3′
pklr	Fw:5′TCATGTTGTCTGGAGAGACTGC 3′
	Rv 5′GAT​TGT​AGA​TCG​CTG​CCT​CAG 3′

### COX/SDH assay

Uninjured and transected animals (6 hpt, 1dpt, and 2dpt) were anesthetized and sacrificed. The stomach and part of the tail were removed to obtain the correct positioning of the animal. Using micro-surgical forceps, excess water was blotted off with absorbent paper. The animal was then coated in Optimal Cutting Temperature (O.C.T.) compound and placed on a cork for quickly freezing the tissues. For the freezing procedure, already published protocols were adjusted (Meng et al., 2014; Kumar et al., 2015). Briefly, liquid nitrogen was placed in a container designed for handling extreme temperatures, and a stainless-steel cup with Isopentane (2-methylbutane) was submerged. The Isopentane was left to cool for a few minutes until about 30% of it had frozen, and the cork was immersed in Isopentane for 25 s until fully frozen, then rested on dry ice for 30 min to aid in the evaporation of Isopentane and maintaining the temperature, preventing thawing or artifacts. Later, the sample was wrapped in aluminum foil to prevent crystal formation and stored at −80°C with dry ice. Longitudinal 10 µm sections were obtained using a Leica CM1850 cryostat. Finally, for the COX and SDH histochemistry, an already published protocol was adjusted (Ross, 2011). Briefly, the slides containing the spinal cord sections were thawed for 30 min at RT, incubated with 1X DAB, 8 M cytochrome C in 0.1 M PBS, 2 mg/mL catalase, 220 mM sucrose for 2 h at RT, washed with distilled water, and incubated with 1.2 mM PMS, 1.6 mM NTB, 1 M sodium succinate, sodium azide/EDTA/PO4 (1.9 mM sodium azide, 1.5 mM EDTA 32.5 mM NaH2PO4·H2O, 217 mM Na2HPO4 ·7H2O) for 30 min at 37°C, and washed with distilled water. Later, the samples were fixed with 10% formalin for 10 min and mounted with a pre-warmed glycerin gelatin solution. 1.25 mM sodium azide was used as a negative control for COX histochemistry, and succinate was replaced by malate as a negative control for SDH. Images were obtained using an inverted confocal microscope Nikon spectral ECLIPSE (C2Si), with a 40X objective.

### Image and statistical analysis

Image acquisition and analysis were performed using Fiji (ImageJ) software. Statistical analyses were conducted using GraphPad Prism 8 software. A one-way ANOVA followed by Dunnett’s multiple comparison test was used to determine the significance of multiple comparisons of normally distributed data. For nonparametric multiple comparisons, the Kruskal–Wallis test, followed by Dunn’s multiple comparison test, was employed. To compare the mean of log2-transformed measurements to a single control value (considered as 0 for non-fold-change data), a one-sample *t*-test was applied. Statistical significance was considered as follows: ****(p < 0.0001); ***(p < 0.001); **(p < 0.01); *(p < 0.05); ns = not significant (not statistically different).

## Results

### Mitochondria localize primarily to the NSPCs with an apical distribution

**FIGURE 1:**
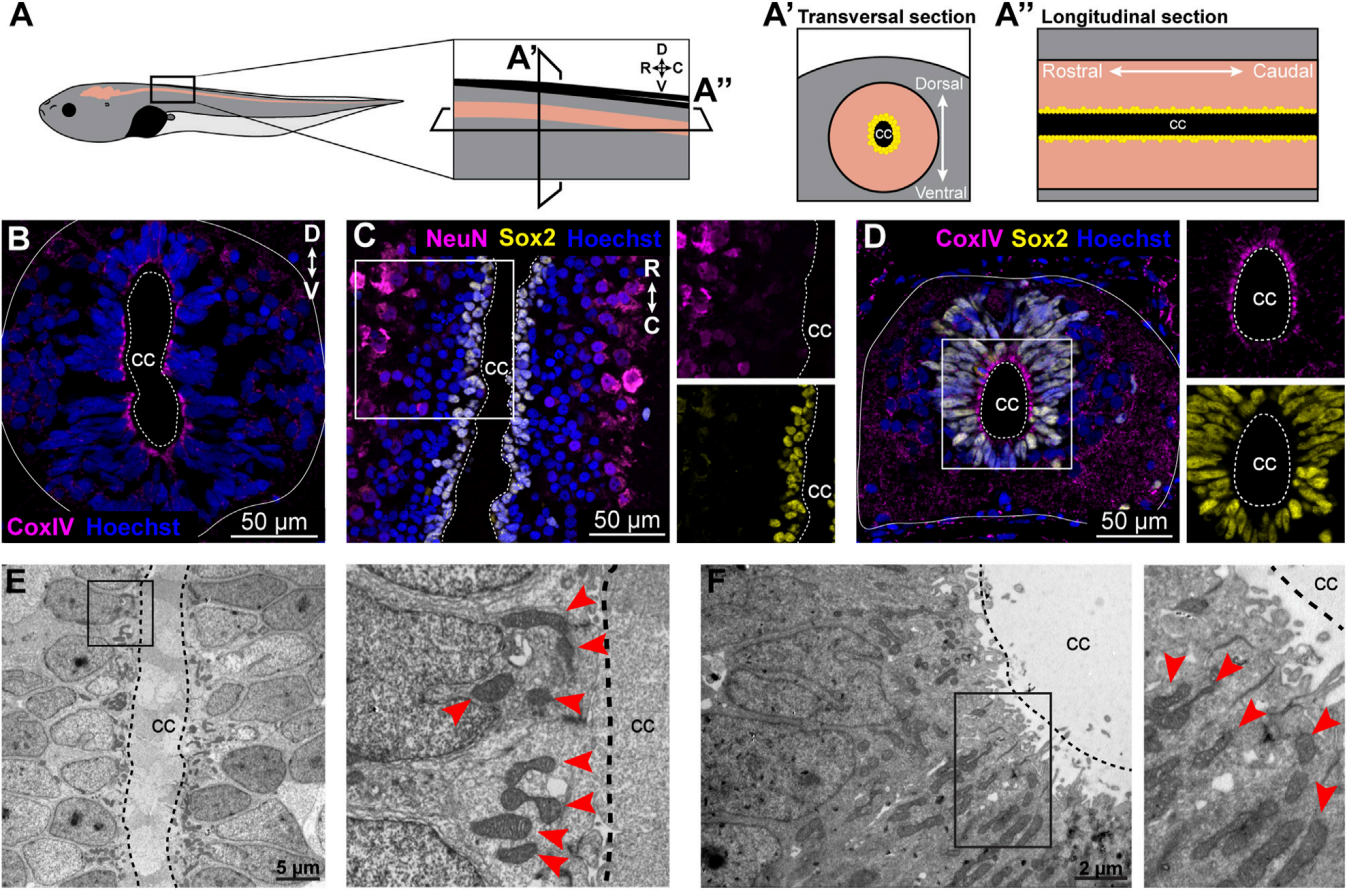
Mitochondria localize primarily to the NSPCs with an apical distribution. **(A)** Illustrative representation of histological sections of *X. laevis* tadpole spinal cord. On the left an NF 50–51 tadpole with the brain and spinal cord in beige. **(A’)** Maximization of a transversal section of the spinal cord with dorsal on top and rostral at the bottom. **(A’’)** Maximization of a longitudinal section of the spinal cord from rostral to caudal. **(B–D)** Immunostainings on transversal **(B, D)** and longitudinal **(C)** spinal cord sections of uninjured animals. Scale bar: 50 µm. **(E–F)** Electron microscopy images on longitudinal **(E)** and transversal **(F)** spinal cord sections of uninjured animals. Scale bar: 5 and 2 μm, respectively. Magnifications of the boxed regions of interest are shown to the right of the corresponding panels. Red arrows indicate mitochondria. CC, Central canal. The continuous line limits the spinal cord, and the dashed line limits the central canal.

We first sought to characterize the localization of mitochondria within the spinal cord in the regenerative stages (R-stages) of *Xenopus laevis*. Spinal cord transversal cryosections were obtained (Figure 1A’) and immunodetection of the mitochondria utilizing the mitochondrial marker CoxIV was performed. Mitochondria were observed mainly in the region adjacent to the central canal (Figure 1B). The spinal cord is composed primarily of NSPCs surrounding the central canal (Muñoz et al., 2015; Edwards-Faret et al., 2018), and astrocytes and neurons are also present in the gray matter (Miller and Liuzzi, 1986; Maier and Miller, 1995). Therefore, spinal cord longitudinal cryosections were obtained (Figure 1A). Immunodetection of NSPC (Sox2+) and neurons (NeuN) was performed, showing that Sox2+ cells correspond to a couple of layers of cells localized surrounding the spinal cord central canal. In contrast, NeuN + cells utilize a more extended region of the spinal cord gray matter but are localized further from the central canal (Figure 1C). Therefore, co-detection of CoxIV and Sox2 was performed, confirming that most of the mitochondria present in the spinal cord are localized in the NSPCs, presenting an apical distribution (Figure 1D). The localization of the mitochondria in the cells surrounding the central canal and the apical distribution of the mitochondria in the spinal cord was confirmed by electron microscopy of longitudinal (Figure 1E) and transversal (Figure 1F) sections.

### SCI drives changes in mitochondria number and localization on NSPCs

**FIGURE 2:**
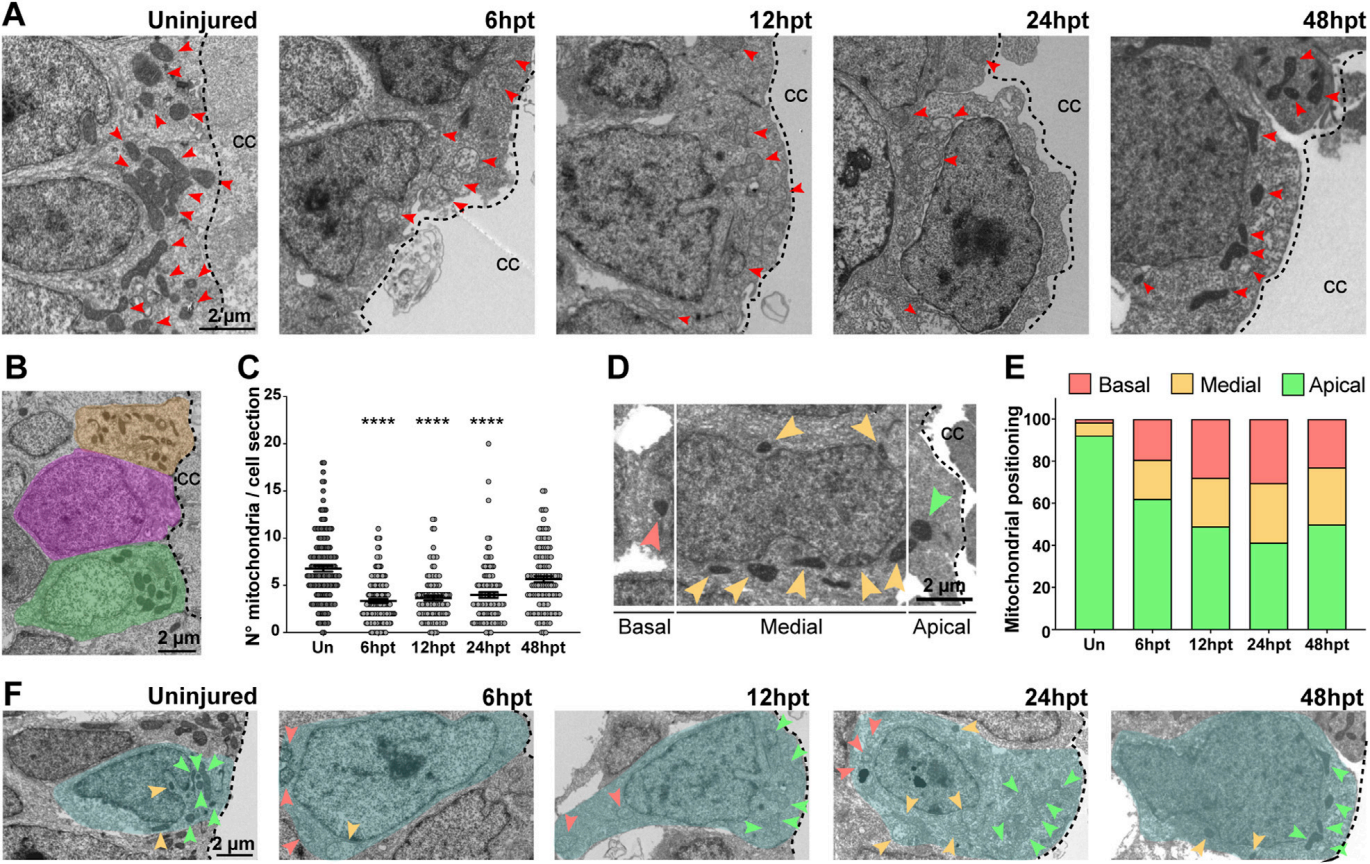
SCI drives changes in mitochondria number and localization on NSPCs. **(A)** Representative electron microscopy images depicting the mitochondria in NSPCs lining the central canal (CC), in uninjured animals and at different times after transection. Red arrows point to individual mitochondria. Scale bar: 2 µm. **(B)** Electron microscopy image with masks highlighting cell sections in different colors. Scale bar: 2 µm. **(C)** Comparison of the number of mitochondria per cell section in CC-lining cells in the uninjured (Un) spinal cord and at different time points after SCI, in hours post-transection (hpt). ****(p < 0.0001), from a Kruskal–Wallis test, followed by Dunn’s multiple comparison test. **(D)** Electron microscopy image representing the criteria used to define the localization of mitochondria within a cell. Arrows point to individual mitochondria present in the apical (green), medial (orange), or basal (coral) section of the cell. Scale bar: 2 µm. **(E)** Percentage of mitochondria localized to the apical, medial, or basal section in the cells lining the central canal before and after SCI. **(F)** Electron microscopy images with masks indicating the cellular area and arrows pointing to individual mitochondria localizing to the apical (green), medial (orange), or basal (coral) section of the cell. Scale bar: 2 µm. Dashed lines limit the central canal and the NSPCs. hpt, hours post-transection.

The number and localization of mitochondria within cells are typically regulated based on cellular requirements. Therefore, mitochondria from cells surrounding the central canal, which correspond to NSPCs, were analyzed in longitudinal spinal cord cryosections in uninjured control animals and at specific time points post-transection (6, 12, 24, and 48 h). Time points for analysis were selected based on prior findings and published data. Lee-Liu et al. (2014) showed that at 24 hpt, metabolic transcript levels are most regulated. Additionally, Peñailillo et al. (2021) reported early transcript regulation related to nutrient response starting 1–2 h post-transection (hpt), with elevated levels persisting until 8 h. Even more, cellular events influenced by mitochondrial and metabolic activity, such as cell death, proliferation, and immune response, were primarily observed between 6–48 hpt. Therefore, the analysis began at 6 hpt, coinciding with the first wave of apoptosis in the spinal cord, a process involving mitochondria (Liu et al., 1997), and continued until 48 hpt. By this time, metabolic transcript levels, which are most regulated at 24 hpt (Lee-Liu et al., 2014), were expected to influence mitochondrial characteristics (Figure 2A). The number of mitochondria was quantified in each cell section adjacent to the central canal. A region of interest (ROI) was defined by tracing the plasma membrane of cells in contact with the central canal (Figure 2B), and mitochondria within the ROI were counted. Cells partially visible in the image were excluded. The number of mitochondria per cell section temporarily decreased, from 6 to 24 hpt. Basal levels were recovered at 48 hpt (Figure 2C). Additionally, each cell adjacent to the central canal was divided into three sections: apical, from the central canal to the beginning of the nucleus; medial, considering all the area occupied by the nucleus; and basal, from the end of the nucleus to the inner plasma membrane, and the localization of the mitochondria was analyzed (Figure 2D). Uninjured animals present 92% of the mitochondria in the apical zone, while redistribution of the mitochondria was observed at all time points after transection, showing nearly 50% of the mitochondria in the apical zone, and the other 50% redistributed almost equally towards the medial and basal zone (Figures 2E, F).

### Mitochondrial morphology and phenotype are altered after SCI

**FIGURE 3:**
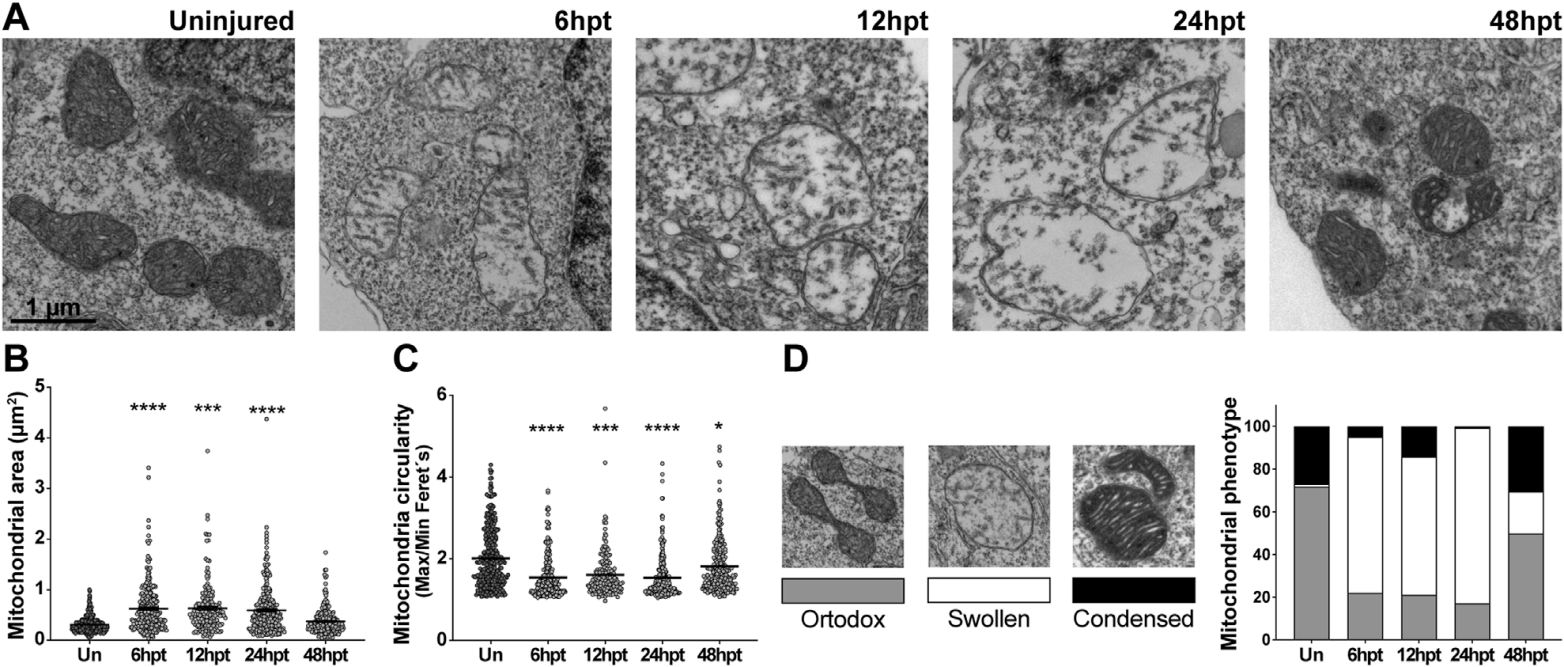
Mitochondrial morphology and phenotype are altered after SCI. **(A)** Representative electron microscopy images depicting the mitochondria in NSPCs lining the central canal in uninjured animals and at different times after transection. Scale bar: 1 μm **(B)** Comparison of the mitochondrial area in the uninjured spinal cord and at different time points after SCI. **(C)** Comparison of the mitochondrial circularity in the uninjured spinal cord and at different time points after SCI. ****(p < 0.0001); ***(p < 0.001); *(p < 0.05), from a Kruskal–Wallis test, followed by Dunn’s multiple comparison test **(B–C)**. (**(D)**, left) Representative images of the different mitochondrial phenotypes evaluated, with the corresponding bar color used for the graph on the right. (**(D)**, right) Changes in mitochondrial phenotype before and after SCI. hpt: hours post-transection.

Mitochondria are dynamic organelles that adjust their morphology (Wai and Langer, 2016) and phenotype (Hackenbrock, 1966) in response to environmental changes and metabolic demands. To assess these adaptations, the mitochondrial area, circularity, and phenotype were analyzed in electron microscope images at specific time points post-transection (6, 12, 24, and 48 hpt) (Figure 3A). An increase in mitochondrial area was observed starting at 6 hpt, which returned to baseline by 48 hpt (Figure 3B), with the mitochondrial area approximately doubling from ∼0.3 µm^2^ to ∼0.6 µm^2^. Mitochondrial circularity can be determined by the ratio of the minimum Feret diameter to the maximum Feret diameter (Max/Min Feret’s), where values closest to 1 represent an increase in circularity. An increase in mitochondrial circularity was observed starting at 6 hpt, which returned to baseline by 48 hpt (Figure 3C), with a change in the mitochondrial Max/Min Feret´s from ∼2.0 to ∼1.5. Mitochondria were classified into three different phenotypes: orthodox, condensed, and swollen, based on previous work (Hackenbrock, 1966). Orthodox mitochondria present less defined cristae with intermediate matrix density; the condensed phenotype presents increased cristae width, with more defined cristae and intermediate matrix density; and the swollen phenotype presents a deployed internal membrane, remnant tubular cristae, dispersed matrix granules responsible for a decrease in electron density, and a two to three-fold size increase (Figure 3D). Mitochondria in uninjured animals are mostly orthodox ∼72%, while ∼27% show a condensed phenotype, and only ∼1% a swollen phenotype (Figure 3D). The mitochondria change their phenotype starting at 6 hpt and return to levels similar to the uninjured animals at 48 hpt, an increase in the swollen phenotype from ∼1 to 70%–80%, and a decrease in the orthodox phenotype from ∼72 to ∼20%, was observed.

### Mitochondrial fission transcript levels are transitory elevated after SCI

**FIGURE 4:**
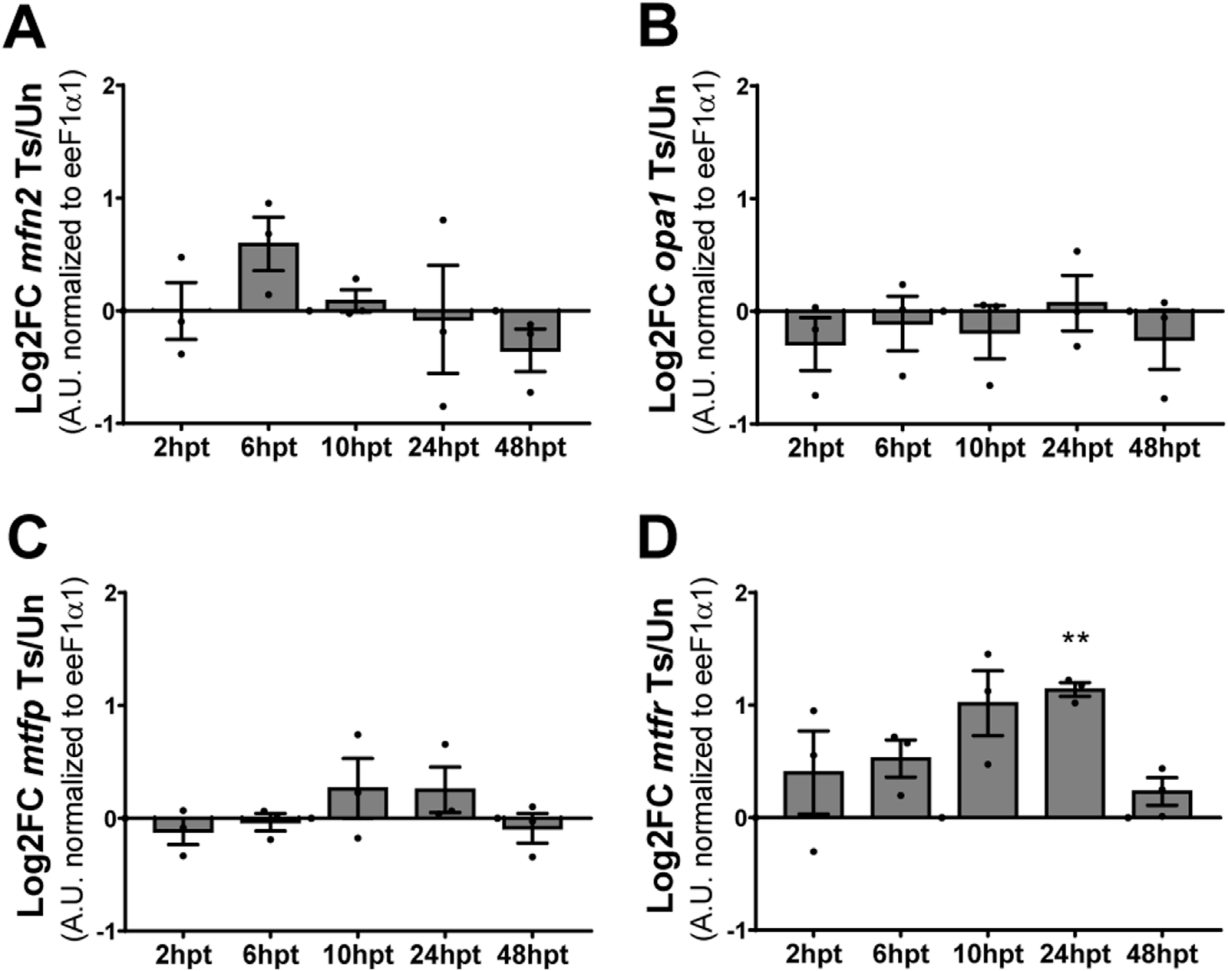
Mitochondrial fission transcript levels are transitory elevated after SCI. **(A, B)** Changes in transcript levels of mitochondrial fusion-related proteins *mfn2* **(A)** and *opa1* **(B)**. **(C, D)** Changes in transcript levels of mitochondrial fission-related proteins *mtfp* **(C)** and *mtfr* **(D)**. All values are normalized to eeF1α1. **(p < 0.01), from a one-sample *t*-test. Log2FC: Logarithm base 2 of Fold-Change. Ts/Un, Transected over Uninjured. A. U, Arbitrary Units.

Mitochondrial morphology results from a balance between two dynamic and opposing processes: mitochondrial fusion and fission. The morphology changes when the balance between these processes is altered (Tilokani et al., 2018; Chan, 2020). Since a change in mitochondrial morphology was observed after SCI, fusion- and fission-related transcript levels were analyzed by RT-qPCR at specific time points post-transection (2, 6, 10, 24, and 48 hpt). The protein Optic Atrophy 1 (Opa-1), a GTPase of the dynamin family, participates in the fusion of the inner mitochondrial membrane, and Mitofusin 2 (Mfn2), a transmembrane GTPase participates in the fusion of the outer mitochondrial membrane, while the protein Mitochondrial Fission Process 1 (Mtfp1) is localized at the inner mitochondrial membrane, and Mitochondrial fission regulator 1 (Mtfr1) localized in the outer mitochondrial membrane and is involved in the process of mitochondrial fission (Wai and Langer, 2016; Tilokani et al., 2018; Chan, 2020). Therefore, the transcript levels of *mfn2* and *opa1*, and *mtfp1* and *mtfr1* were analyzed as a readout of mitochondrial fusion and fission processes regulation, respectively. *Mfn2* (Figure 4A), *opa1* (Figure 4B), and *mtfp* (Figure 4C) transcript levels remained unaltered during all the analyzed time points, while *mtfr* presented an acute and transient increase in the transcript levels at 24 hpt (Figure 4D).

### Mitochondrial function and cellular energy balance are maintained after SCI

**FIGURE 5:**
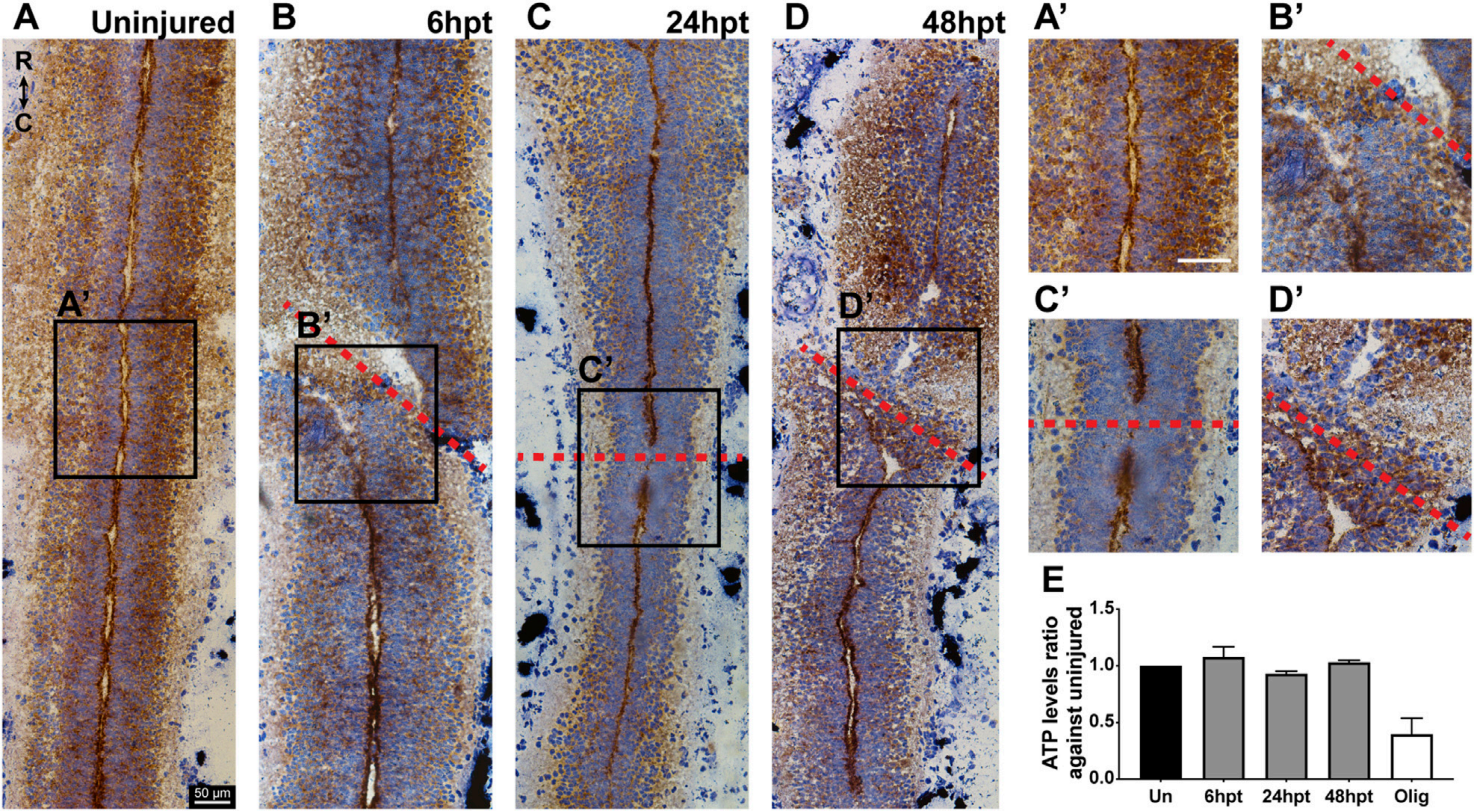
Mitochondrial function and ATP production are maintained after SCI. **(A–D)** Representative bright field images of cytochrome c oxidase (COX) and succinate dehydrogenase (SDH) histochemistry on longitudinal cryosections of the uninjured spinal cord **(A)** and at different time points after SCI **(B–D)**. **(A’–D’)** Magnifications of the boxed regions of interest on **(A–D)**. Red dashed lines mark the site of the transection. COX: brown staining. SDH: bluish-purple staining. R → C: rostral to caudal orientation. Scale bars: 50 μm **(E)** Comparison of ATP levels in the uninjured spinal cord and at different time points after SCI. hpt: hours post-transection. Kruskal–Wallis test, followed by Dunn’s multiple comparison test was performed.

Mitochondrial phenotype changes depending on the energy requirement of the cell. Orthodox phenotype is observed generally in intact tissue and is related to slow substrate consumption and low respiratory rate, while the condensed phenotype is observed when mitochondria activate oxidative phosphorylation and present a high respiratory rate, and the swollen phenotype is observed when no oxidative phosphorylation is occurring (Hackenbrock, 1966). It has been described that the mitochondrial swollen phenotype could be linked to mitochondrial uncoupling, where mitochondria might alter their phenotype under conditions that affect oxidative phosphorylation. However, if the mitochondrial organization were to reach a point where the swollen phenotype became irreversible and phosphorylation integrity was lost, the mitochondria would be considered damaged (Hackenbrock, 1966). Given that the swollen phenotype was observed following SCI, a Cytochrome C Oxidase/Succinate Dehydrogenase (COX/SDH) double-labeling histochemistry was conducted to assess whether this phenotype change is associated with mitochondrial dysfunction. The catalytic subunits of COX are encoded by mitochondrial DNA; therefore, COX assembly and activity are dependent on functional mitochondria with intact DNA. While SDH is encoded by nuclear DNA; therefore, SDH activity is not dependent on mitochondria integrity (Ross, 2011). At the histological level, in the presence of mitochondrial dysfunction, double staining with the COX/SDH activity reveals cells that are positive for SDH, appearing in a bluish-purple color, with minimal or no brown COX staining (Ross, 2011). In longitudinal cryosections of uninjured animals, the cell layers closest to the central canal present a coloration with a predominant bluish-purple color and a brown color concentrated in the apical zone of the cells surrounding the central canal, coincident with the localization of mitochondria (Figures 1D, E, 5A, A’). This characteristic distribution was mostly maintained after SCI at all time points analyzed (Figures 5B–D, B’–D’). Thus, the activity of COX and SDH remained stable upon spinal cord injury. Additionally, the cellular energy level was evaluated by measuring ATP levels, which is indicative of metabolic active cells. ATP levels remained unchanged after SCI at all analyzed time points (Figure 5E). These results suggest that minimal changes in mitochondria function are observed after SCI in *X. laevis* regenerative stages.

### SCI selectively and transiently decreases mitochondrial membrane potential in NSPCs

**FIGURE 6:**
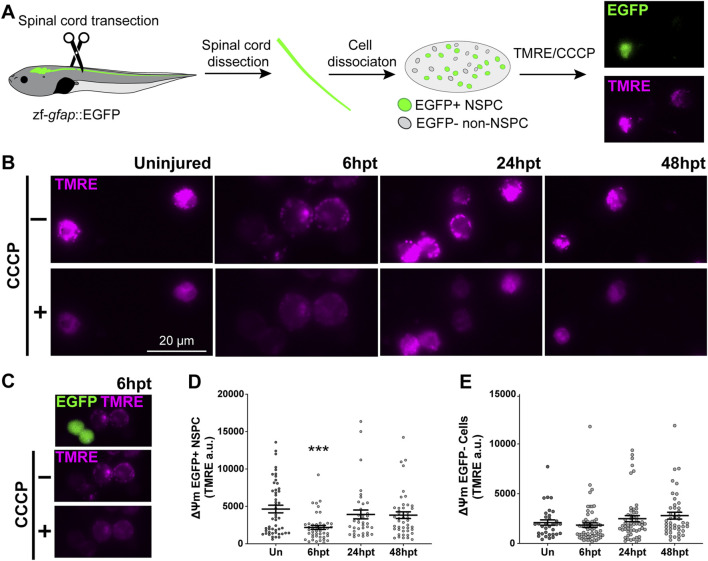
SCI selectively decreases mitochondrial membrane potential in NSPCs. **(A)** Illustrative representation of experimental procedure: NF 50–51 zf-*gfap*::EGFP transgenic tadpoles expressing EGFP in neural stem progenitor cells (NSPCs) were subjected to complete spinal cord transection. Spinal cords of uninjured (Un) or injured tadpoles (at 6, 24, and 48 hpt) were collected, dissociated, and plated. NSPCs are differentiated from the non-NSPCs by the EGFP fluorescence. Once plated, cells were treated with the fluorescent dye TMRE, followed by the mitochondrial uncoupler CCCP. Cells were imaged before and after the CCCP administration to obtain the resting ΔΨm. **(B)** Representative images of TMRE signal before (−) and after (+) CCCP administration in plated cells collected from Un spinal cords, or at 6, 24, and 48 hpt. Scale bar: 20 μm **(C)** Representative images of cells collected at 6hpt, the merge of TMRE and EGFP signals are shown at the top, followed by the TMRE channel before and after CCCP administration. **(D–E)** Changes in ΔΨm of EGFP + NSPC **(D)** and EGFP- Cells **(E)**. ΔΨm: mitochondrial membrane potential. ***(p < 0.001), from a One-way ANOVA, followed by Dunnett’s multiple comparison test.

The results from the previous section showed that the observed mitochondrial swollen phenotype did not result in mitochondrial damage, prompting us to test whether this phenotype was associated with mitochondrial uncoupling. A characteristic of mitochondrial uncoupling is a decrease in the mitochondrial membrane potential (Δψm). However, this effect is generally transient, as a prolonged decrease could lead to pathological consequences (Zorova et al., 2018). Since the mitochondrial swollen phenotype was observed in cells surrounding the spinal cord central canal, which correspond to NSPCs, the mitochondrial membrane potential was evaluated in NSPCs and non-NSPCs within the spinal cord of uninjured and transected animals at defined time points post-transection (6, 24, and 48 hpt). The *Xenopus laevis* Xla. Tg (Dre.gfap:EGFP)^Larra^ transgenic line was used, as it expresses GFP under an NSPC-specific promoter (Edwards-Faret et al., 2021), allowing for the identification of NSPCs GFP+ and non-NSPCs GFP- cells (Figure 6A). At the defined time points, the spinal cord was dissected, cells were dissociated and plated on a coverslip. Cells were then treated with tetramethylrhodamine (TMRE), a dye that accumulates in the mitochondria in a Δψm-dependent manner. Protonophores such as Carbonyl cyanide m-chlorophenylhydrazone (CCCP) reduce the Δψm and thereby induce the dissipation of TMRE from the mitochondria. The combined use of TMRE and CCCP enables the assessment of Δψm (Figure 6A). A decrease in the Δψm was observed in NSPC GFP+ cells at 6 hpt, returning to basal levels at 24 hpt (Figures 6B–D). At the same time, no changes were detected in non-NSPC GFP+ cells (Figures 6C, E), suggesting that the mitochondrial uncoupling occurs as a specific and transient response of NSPCs to SCI.

### SCI induces a transient switch toward glycolytic metabolism

**FIGURE 7:**
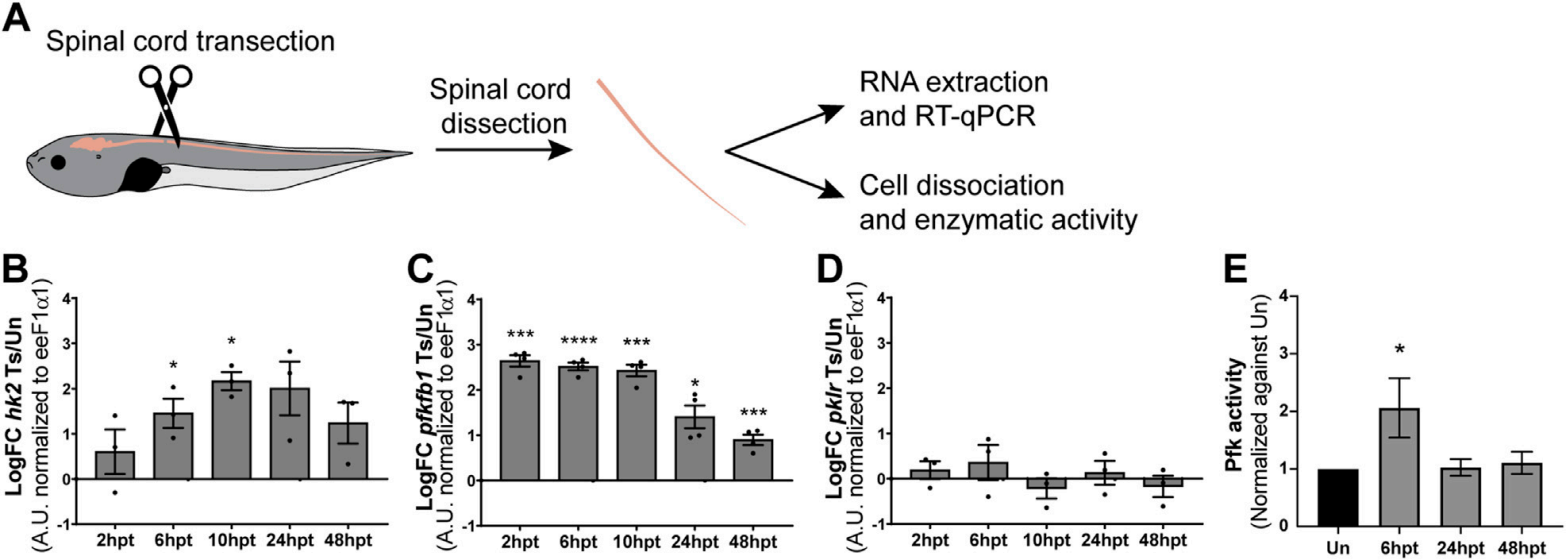
SCI induces a transient switch towards glycolytic metabolism. **(A)** Illustrative representation of experimental procedure: NF50-51 tadpoles were subjected to complete spinal cord transection. Then, spinal cords of uninjured or injured tadpoles at different time points were collected for either RNA extraction and RT-qPCR analysis or cell dissociation and biochemical analysis of enzymatic activity. **(B–D)** Changes in transcript levels of glycolysis-related transcripts *hk2* **(B)**, *pfkfb1* **(C)**, and *pklr* **(D)**. Transcript levels were normalized against eeF1α1. Ts/Un: Transected over Uninjured. ****(p < 0.0001); ***(p < 0.001); *(p < 0.05), from a one-sample *t*-test. **(E)** Changes in the activity levels of the glycolytic enzyme Pfk. *(p < 0.05), from a Kruskal–Wallis test, followed by Dunn’s multiple comparison test.

The Δψm is essential for ATP production; if Δψm is decreased, the mitochondria produce less ATP, resulting in a decrease in the cytosolic ATP/ADP ratio, which enhances glycolysis to maintain the ATP levels in the cell (Maldonado and Lemasters, 2014). As ATP levels were not affected by SCI, but a transient decrease in Δψm was observed, the activation of glycolytic metabolism was evaluated. In glycolysis, three irreversible reactions, catalyzed by the rate-limiting enzymes hexokinase (Hk), phosphofructokinase-1 (Pfk1), and pyruvate kinase (Pkr), regulate the rate of the glycolytic pathway (Chandel, 2021). Additional allosteric regulation of the glycolytic rate occurs by another enzyme: 6-phosphofructo-2-kinase/fructose 2,6-bisphosphatases (Pfkfb), which control the levels of the allosteric activator of Pfk1, fructose 2,6-bisphosphate (Fru-2,6-P2) (Ros and Schulze, 2013). To assess glycolysis regulation, transcript levels of *hk2*, *pfkfb1*, and *pklr* were analyzed by RT-qPCR at specific post-transection time points (2, 6, 10, 24, and 48 hpt) (Figure 7). The transcript levels of *hk2* showed an early and transient increase from 6 to 10 hpt (Figure 7B), while *pfkfb1* transcript levels exhibited a sustained increase across all analyzed time points (Figure 7C). In contrast, *pklr* transcript levels remained unchanged (Figure 7D). It is important to consider that changes in cellular metabolism are often rapid and dynamic. Therefore, glycolytic activity was confirmed by measuring Pfk enzymatic activity. A fast and transient increase in Pfk activity was observed at 6 hpt, with levels returning to baseline by 24 hpt (Figure 7E). This together allows us to suggest that there is an early increase in the glycolytic rate on the spinal cord after SCI.

## Discussion

In this study, we employed electron and confocal microscopy to characterize the temporal mitochondrial response in wild-type and transgenic *Xenopus laevis* lines during spinal cord regeneration. Herein, we described that mitochondria present a rapid response after SCI. Mitochondria are expressed mainly in the apical region of the NSPCs surrounding the spinal cord central canal, a disposition that is lost after SCI. Additionally, a decrease in the number of mitochondria per cell section is observed, accompanied by an increase in the mitochondrial area and circularity. SCI also induced a phenotypic change in mitochondria, with a preference for a swollen morphology, which does not compromise mitochondrial function, as indicated by COX/SDH staining, and maintained cellular energy balance. Furthermore, this morphological change is accompanied by an early and transient decline in mitochondrial activity, reflected by a decrease in NSPCs mitochondrial membrane potential and a shift toward glycolytic metabolism, as evidenced by increased transcript levels and enzyme activity of glycolytic genes. Therefore, our results suggest a transient metabolic shift toward glycolysis might be necessary during spinal cord regeneration.

In uninjured animals, mitochondrial markers were expressed mainly in the NSPCs surrounding the *Xenopus laevis* spinal cord central canal, which is consistent with the observations in other animal models, such as zebrafish (Ogino et al., 2016) and macaque (Alfaro-Cervello et al., 2014). Additionally, the number and localization of the mitochondria within cells are usually regulated depending on the cellular requirements. For example, mitochondria are concentrated in the leading process of migratory adult NSPCs but change their localization towards a perinuclear distribution upon differentiation (Kim et al., 2015). We observed an apical distribution of the mitochondria in the cells surrounding the central canal, an area that is demanding in energy, as the ciliary roots are localized there, and the ciliary beating for promoting the flow of cerebrospinal fluid (CSF) through the central canal is ATP-dependent (Mirzadeh et al., 2010). After SCI, a change in the mitochondrial distribution was observed, this could be explained by changes in the cellular energy requirements, as during at least the first 24 hpt extensive transcriptome changes occur (Lee-Liu et al., 2014; Peñailillo et al., 2021), while the cerebrospinal fluid barrier is affected, and the damaged cells may not be able to regulate the CSF transport.

Herein, we observed a decrease in mitochondrial number and an increase in mitochondrial area. This does not appear to be strongly regulated by mitochondrial fusion, as fusion transcript levels remained unchanged over the analyzed period. However, the restoration of the mitochondrial number correlates temporarily with an increase in fission-related *mfpr* transcript levels, suggesting that the fission process might be involved. While we cannot rule out a change in mitochondrial mass, the decrease in mitochondrial area may reflect mitochondrial relocalization instead. Surrounding the spinal cord’s central canal are some cell sections with no observable nuclei, indicating that they are cellular projections either from other NSPCs or cells located in deeper layers (Muñoz et al., 2015; Edwards-Faret et al., 2018). We found that these nucleus-free sections often contain mitochondria, and, as we also observed that there is a change in mitochondrial localization within the cell, the apparent reduction in mitochondria per cell section may result from a redistribution of mitochondria out of these projections or into NSPC projections outside the analyzed plane. However, we cannot discard the role of mitochondrial fusion, as transcript levels do not always predict the protein levels or function. Furthermore, mitochondrial swelling is characterized by an expanded, diluted matrix, with most mitochondria becoming 3 to 4 times larger than in their pre-swollen state (Hackenbrock, 1966). Our observation of transient mitochondrial swelling, aligned with the temporary increase in mitochondrial area, likely reflects an additional aspect of this mitochondrial phenotype shift.

High-throughput analyses have shown changes in transcripts related to cellular metabolism that are conserved during different regenerative processes. For example, *Xenopus tropicalis* (Love et al., 2011; Patel et al., 2022) and zebrafish (Sinclair et al., 2021) tail regeneration involves an epimorphic regeneration, meaning that the regenerative process involves the formation of a blastema or regeneration bud, and *Xenopus laevis* spinal cord regeneration consists of neoblastic regeneration and considers the proliferation and differentiation of stem cells (Lee-Liu et al., 2014; Lee-Liu et al., 2018). All these cases suggest an increase in glucose uptake and/or metabolic switch towards aerobic glycolysis, the Warburg effect, which is characterized by a high glucose uptake rate and high lactate production, accompanied by functional mitochondria and oxidative phosphorylation (DeBerardinis and Chandel, 2020). Additionally, Hydra head regeneration involves a morphallaxis regeneration, meaning that the regenerative process involves reorganizing existing tissues with almost no cell proliferation, and it has been shown that during regeneration, an increase in glycolysis is observed as well (Osuma et al., 2018). Even though the RNAseq analyses have provided important information regarding the cellular metabolic state during regeneration, it is necessary to consider that changes at the transcriptomic level reflect a slow and sustained response over time; they will not necessarily reflect dynamic and transient changes, which generally characterize changes in cellular metabolism. In zebrafish, it has already been demonstrated that the switch to aerobic glycolytic metabolism is necessary for the formation of blastema and tail regeneration, as the blockade of the glucose metabolism with the glucose analog 2-deoxyglucose affects the glycosylation of proteins necessary for the blastema formation (Sinclair et al., 2021), and lactate production (Scott et al., 2022), and thus, blocking regeneration. But, to date, analysis of the mitochondrial response and cellular metabolism during *Xenopus laevis* spinal cord regeneration was not available, this is the first study relating temporal mitochondrial ultrastructural data with mitochondrial function and directly showing an increase in the glycolytic metabolism during spinal cord regeneration, supporting this metabolic switch as a hallmark of a successful regenerative process.

Previous studies show that the SCI mechanical damage generates cell death and that the biochemical response is propagated, leading to further cell loss and extending the damage. Cell death is quickly regulated post-injury in animals with regenerative abilities, regardless of their regeneration strategy. For example, Hydra shows nearly no cell death at 16 h post-head amputation (Chera et al., 2009). Conversely, zebrafish and *Xenopus laevis* show a peak of cell death at 24 h post-tail amputation that drops in the following days (Tseng et al., 2007; Hui et al., 2010). In *Xenopus*, if this cell death response is prolonged, it disrupts the regenerative process (Tseng et al., 2007), highlighting the importance of a well-timed and spatially regulated response. In line with this, the polarization state of the mitochondrial membrane plays a crucial role in determining outcomes related to excitotoxicity and oxidative damage, the two leading causes of cell death in SCI (Slater et al., 2022a). Both mitochondrial calcium uptake during excitotoxicity (Stout et al., 1998) and ROS production (Hansford et al., 1997), depend on mitochondrial membrane potential. Mild mitochondrial uncoupling has shown a protective effect in models of both excitotoxicity (Stout et al., 1998) and SCI (Jin et al., 2004). Herein, we observed that there is a change in the mitochondrial phenotype, characterized by an increase in the swollen phenotype, which is related to mitochondrial uncoupling (Hackenbrock, 1966), which is accompanied by a transitory decrease in the mitochondrial membrane potential and an increase in glycolysis, evidenced by elevated glycolytic transcripts and increased PFK-1 enzymatic activity, hallmarks of mitochondrial uncoupling. The increase in mitochondrial swollen phenotype persists until 24 hpt, which correlates with a negative regulation of pro-apoptotic transcript levels (Lee-Liu et al., 2014) and a positive regulation of anti-apoptotic protein levels (Lee-Liu et al., 2018). These findings suggest that exploring the role of mitochondrial uncoupling in cell death and protection following SCI could provide valuable insights.

Additionally, *Xenopus laevis* regenerative stages present NSPCs surrounding the spinal cord central canal (Edwards-Faret et al., 2021), which respond quickly with an increase in proliferation (Muñoz et al., 2015; Edwards-Faret et al., 2021), generating new NSPC-like cells that participate in the stump closure and eventually allowing the continuity of the central canal (Muñoz et al., 2015), and the generation of new neurons (Muñoz et al., 2015; Edwards-Faret et al., 2021), to restore lost connections and recovery of the swimming capacity. Highly proliferative cells are characterized by shifting their metabolism to aerobic glycolysis (DeBerardinis and Chandel, 2020), which is essential for activating pathways that support the synthesis of cellular building blocks (Lunt and Vander Heiden, 2011; Liberti and Locasale, 2016). Furthermore, high lactate production can enhance proliferation by modifying cell cycle regulatory proteins (Liu et al., 2023). Additionally, mTOR is thought to regulate and be regulated by glycolytic metabolism (Ito and Suda, 2014). It has been described that mTORC1 is rapidly and transiently activated following SCI, which is necessary for NSPC proliferation (Peñailillo et al., 2021). The temporality of mTORC1 regulation coincides with the glycolytic activation described in the present work, which precedes the peak of NSPCs proliferation. Therefore, it would be interesting to determine whether this metabolic switch regulates NSPCs proliferation during spinal cord regeneration.

Finally, a shift to oxidative phosphorylation has been linked to stem cell differentiation (Vieira et al., 2011), as specific glycolytic or tricarboxylic acid cycle metabolites can regulate post-translational modifications in proteins that drive specific differentiation programs (Tarazona and Pourquié, 2020). During spinal cord regeneration, some NSPCs differentiate into neurons (Muñoz et al., 2015), and neurogenic markers transcript levels are increased early after SCI (Lee-Liu et al., 2014), which is preceded by the return to basal glycolytic activity. Therefore, it would be interesting to test if the transience in the glycolytic switch is necessary for the neurogenesis event during spinal cord regeneration.

In conclusion, our study provides a detailed temporal analysis of mitochondrial dynamics and metabolic changes during spinal cord regeneration in *Xenopus laevis*. The rapid increase in mitochondrial swelling and shift towards glycolysis, followed by a return to basal levels, suggests a tightly regulated metabolic response. Furthermore, the conserved metabolic responses observed across different regenerative models, such as aerobic glycolysis in blastema formation and tissue reorganization, underscore the importance of cellular metabolism in facilitating regeneration. Future research investigating the exact role of mitochondrial dynamics and metabolic shifts on NSPC survival, proliferation, and differentiation will be vital to further our understanding of the molecular basis of spinal cord regeneration. This knowledge could open pathways for developing therapeutic strategies to enhance regeneration following spinal cord injuries.

## Data Availability

The original contributions presented in the study are included in the article/supplementary material, further inquiries can be directed to the corresponding author.
